# Manifold transform by recurrent cortical circuit enhances robust encoding of familiar stimuli

**DOI:** 10.1371/journal.pcbi.1013587

**Published:** 2025-10-24

**Authors:** Weifan Wang, Xueyan Niu, Liyuan Liang, Tai-Sing Lee

**Affiliations:** 1 Center for the Neural Basis of Cognition, Carnegie Mellon University, Pittsburgh, Pennsylvania, United States of America; 2 Neuroscience Institute, Carnegie Mellon University, Pittsburgh, Pennsylvania, United States of America; 3 Computer Science Department, Carnegie Mellon University, Pittsburgh, Pennsylvania, United States of America; UT Austin: The University of Texas at Austin, UNITED STATES OF AMERICA

## Abstract

A ubiquitous phenomenon observed along the ventral stream of the primate hierarchical visual system is the suppression of neural responses to familiar stimuli at the population level. The observation of the suppression of the neural response in the early visual cortex (V1 and V2) to familiar stimuli that are multiple times larger in size than the receptive fields of individual neurons implicates the plausible development of recurrent circuits for encoding these global stimuli. In this work, we investigated the neural mechanisms of familiarity suppression and showed that a recurrent neural circuit based on Hebbian learning, consisting of neurons with small and local receptive fields, can develop to encode specific global familiar stimuli robustly as a result of familiarity training. We proposed that the learned recurrent circuit implements a manifold transform. The recurrent circuit compresses the dimensions of nuisance variations of a familiar image in the neural response manifold relative to the dimensions for discriminating different familiar stimuli, resulting in increased robustness of the global stimulus representation against noise and other irrelevant perturbations. We demonstrate that a recurrent circuit implements the manifold transform using a mixed strategy of locally linear and globally nonlinear computations, where the local linear computation selectively redistributes recurrent gain to enhance concept discrimination. These results provide testable predictions for neurophysiological experiments.

## Author summary

In this research, we explored how the brain can become more efficient at processing familiar visual information. When we repeatedly see something, our brain’s response to it changes. In response to familiar stimuli, neurons across different visual areas of the mammalian visual system become more selective and their overall activities decrease. We developed a computational model to investigate why this happens and what functional advantages these mechanisms might provide. We discovered that familiarity leads to the development of a more efficient and robust neural representation of what we see. It allows us to rapidly and robustly recognize our friend’s face despite changes in lighting conditions, view angle, or facial expression. Our model showed that through repeated exposure, the brain’s neural circuits, even in the early stages of visual processing, rapidly adapt and organize themselves to focus on important and consistent features in our visual environment while becoming less sensitive to irrelevant variations, and distractions.

## 1. Introduction

Familiarity suppression refers to a phenomenon observed in the inferotemporal cortex (ITC) [1–7] and more recently in early visual cortex [8] that repeated exposure to a set of familiar visual stimuli leads to the suppression of neural responses to these stimuli, particularly in the later part of the temporal responses. There is evidence in the inferotemporal cortex that familiarity training leads to the sparsification of population neural representation to the familiar stimuli, as neurons’ responses to their preferred familiar stimuli were found to be enhanced, while their responses to non-preferred familiar stimuli were suppressed, resulting in a sharpening of the stimulus selectivity tuning curves of the neurons [6,7].

In the early visual cortex, Huang et al. [8] showed that neurons with localized receptive fields became sensitive to the global context of familiar images. Based on timing, it can be inferred that this sensitivity is mediated by the recurrent circuits within V2 rather than feedback from higher visual areas. Similar effects have also been observed in V1 as well, but with a shorter delay with stimulus onset, significantly earlier than the familiarity effects in IT. These observations suggest a rapid plasticity mechanism in the early visual cortex modifying the recurrent circuit within each visual area along the visual hierarchical system to encode global or semi-global familiar image context. These findings suggest that neurons in the early visual cortex, with local receptive fields, can rapidly learn recurrent excitatory circuits to encode global images.

The computational rationale and neural mechanisms of rapid neural plasticity are not well understood, though proposals on its behavioral benefits have focused on image discrimination, reduced saliency, and novelty detection [6,7,9,10]. One promising framework conceptualizes familiarity training as a type of manifold transform. This transform is mediated by recurrent circuits which, through Hebbian learning, encode relationships between local visual concepts. By finding the correct geometric relations between concepts and their variants generated by nuisance transformations (e.g., view angles or occlusions), this process reshapes the representation of global image context to facilitate an invariant representation at the population level [11–14]. In this work, we investigate this proposal.

In this paper, we develop a V1-based neural circuit model based on Hebbian learning and other standard V1 circuitry elements that can account for the familiarity training effects. This is a canonical circuit motif that can be generalized to V2, V4, and IT. We analyzed this circuit to show that familiarity training of the global image stimulus transforms the neural representation manifold in such a way that nuisance variations of the same concept are ignored while distinction of different visual concepts is maintained. We demonstrated that this manifold transform provides a more noise-robust encoding of familiar images or concepts. Our findings show the recurrent circuit performs manifold transformation using a mixed local-linear and global-nonlinear strategy, depending on signal-to-noise ratio and the training stage, and the local linear strategy redistributes recurrent gain to enhance concept discrimination. This novel perspective on cortical recurrent circuits provides insights into the functional rationales underlying the familiarity learning observed in the various visual areas along the hierarchical visual system.

## 2. Results

### 2.1. Plastic recurrent neural circuit model of primary visual cortex

Familiarity training effects have been reported in macaque ITC and V2 as well as in mouse V1 [10,15,16]. We constructed a neural circuit model of the primary visual cortex to demonstrate that plastic horizontal connections can reproduce familiarity effects based on Hebbian learning mechanisms. Such a retinotopic map-based circuit with basic associative learning mechanisms is likely generalizable to understanding the familiarity effect that is similarly observed in V2, V4, and IT.

#### Connectivity and dynamics.

**Fig 1:**
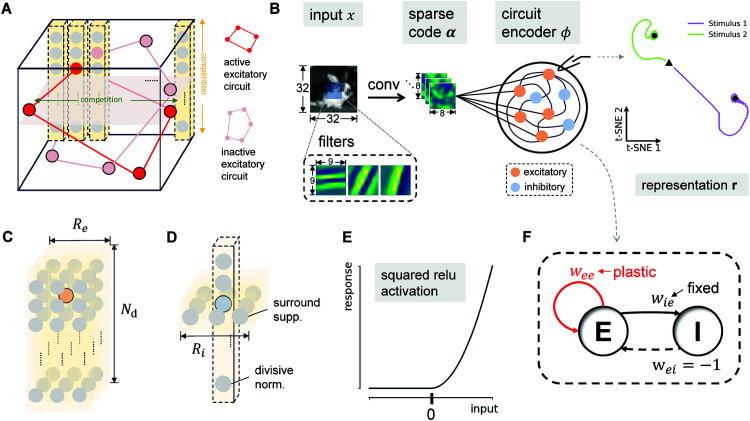
Recurrent circuit model of the primary visual cortex. **(A)** The network architecture. An excitatory sub-circuit, spanning multiple hypercolumns, encodes a global image. Different local subcircuits encode different global images. Neurons compete via two inhibitory mechanisms: (i) suppression among neurons within the same feature channel across different hypercolumns, and (ii) divisive normalization among neurons representing different features within the same hypercolumn. The network input, α, is the sparse code representation of an image [17], and its output, r, is the resulting steady-state response of the excitatory-inhibitory population. **(B)** Computational function. The circuit operates as an attractor network, transforming the sparse code of an input stimulus α that is generated via convolution by a dictionary of filters (lower left: three example filters) into neural representations (r). Right: trajectories of population activity of excitatory neurons to two stimuli (triangle = start, circle = end). Both converge to distinct fixed points, providing stable stimulus representations. The example input image is publicly available here, also see [21]. **(C)** The excitatory neighborhood. For a given target excitatory neuron (orange circle), its neighborhood consists of all excitatory neurons (gray circles) within an $R_{e}\;\times \;R_{e}$ square region, spanning all feature channels. **(D)** The inhibitory neighborhood. For a given target inhibitory neuron (blue circle), its presynaptic neighborhood of excitatory neurons (gray circles) mediates two functions: surround suppression via connections from an $R_{i}\;\times \;R_{i}$ spatial region and divisive normalization via connections from the same hypercolumn. **(E)** The neuronal activation function, which maps total synaptic input to a non-linear firing rate response. **(F)** Connection types and plasticity. The model includes excitatory-to-excitatory connections (E-E, initial weight *w*_*ee*_), excitatory-to-inhibitory connections (E-I, weight *w*_*ie*_), and inhibitory-to-excitatory connections (I-E, weight *w*_*ei*_ = −1). Only E-E connections are plastic.

The network model (Fig 1A) is a firing-rate-based recurrent neural network with *N*_*h*_ hypercolumns (with *N*_*r*_ rows and *N*_*c*_ columns). Each hypercolumn comprises *N*_*d*_ excitatory neurons with receptive fields (RF) derived from sparse coding [17,18]. We have $N_{e}=N_{r}\;\times \;N_{c}\;\times \;N_{d}$ excitatory neurons and the same number (*N*_*i*_) of inhibitory neurons in the network. Each excitatory neuron *k* receives a projection from its excitatory neighborhood (*NE*(*k*)) with a spatial extent of *R*_*e*_, and extends vertically to include feature channels (Fig 1C). The size of the excitatory neighbor is then $|NE(k)|=N_{d}\;\times \;{\left(2R_{e}\;+\;1\right)}^{2}$. Each inhibitory neuron *k* receives projections from the excitatory neurons of the same feature channel located in its inhibitory neighborhood (*NI*(*k*)) with range *R*_*i*_ and projects back to all excitatory neurons in the network, mediating surround suppression. In addition, this inhibitory neuron will receive projections from excitatory neurons within the same hypercolumn, and uniformly inhibits these excitatory neurons in return, as a form of divisive normalization [19,20] (Fig 1D). Therefore, the size of the inhibitory neighbor is $|NI(k)|={\left(2R_{i}+1\right)}^{2}+N_{d}-1$.

The dynamics of the excitatory population and inhibitory population are given as:

$$\tau_{e}\frac{dr_{k}^{e}}{dt}=-r_{k}^{e}+\sigma (\sum_{l}W_{kl}^{ee}\;r_{l}^{e}+\sum_{l^{\prime }}W_{kl^{\prime }}^{ei}\;r_{l^{\prime }}^{i}+\alpha_{k})$$ (1)

$$\tau_{i}\frac{dr_{k}^{i}}{dt}=-r_{k}^{i}+\sigma (\sum_{l}W_{kl}^{ie}\;r_{k}^{e})$$ (2)

where $r_{k}^{e}$, $r_{k}^{i}$ are the firing rates of the *k^th^* excitatory neuron and inhibitory neuron, respectively. $W_{kl}^{ee}$ is the E-E connections from excitatory neuron *l* to excitatory neuron *k*; similarly $W_{kl}^{ie}$ and $W_{ij}^{ei}$ are the E-I and I-E connections; $\alpha_{k}$ is the input to the excitatory neuron *k* obtained via convolutional sparse coding [18]. We used a squared relu activation function $\sigma (z)=\lfloor z\rfloor_{+}^{2}$ (Fig 1E), as introduced in [20]. $\tau_{e}$ and $\tau_{i}$ are the time constants of excitatory and inhibitory neurons, respectively.

The initial value of the E-E connection is set to *w*_*ee*_/|*NE*(*k*)|, within the excitatory neighborhood. For a single inhibitory neuron, the E-I connection to this neuron is uniformly set to a fixed value *w*_*ie*_/|*NI*(*k*)| within its inhibitory neighbors. The I-E connection from this inhibitory neuron to all excitatory neurons is set to the fixed value −1/*N*_*i*_ (Fig 1F). *w*_*ie*_ is set so that normalization within the hypercolumn and iso-orientation (iso-feature) surround suppression across the hypercolumns are strong enough to ensure the stability of the network. This configuration establishes an attractor network at the computational level (Fig 1B). The recurrent circuit acts as an encoder *ϕ*, mapping the sparse code α(x) to a steady-state representation **r**. This is shown by the trajectory (Fig 1B, right): In the trained network, stimulus-evoked activity of excitatory neurons converges to distinct attractors that stably encode each stimulus.

#### Synaptic plasticity.

Here, we are pursuing a minimal circuit mechanism that could reproduce the familiarity effect in neural circuits. Hence, we begin by considering excitatory plasticity only, assuming that inhibitory connectivity remains static and is not subject to plasticity. Hence, only *W^ee^* is subject to associative learning, while *W^ei^* and *W^ie^* are fixed (Fig 1F). Classical familiarity effects are characterized by two concurrent changes in neural activity: a decrease in the population-averaged response, and a selective increase in the activity of a sparse ensemble of neurons highly tuned to the stimulus. This dual effect can be explained by a mechanism similar to the Bienenstock–Cooper–Munro (BCM) learning rule, as inferred by Lim et al. [22]. In BCM learning, a sliding modification threshold determines whether a synapse undergoes depression or potentiation, leading to an increase in the selectivity of neurons for the familiar stimulus. The typical implementation of the BCM rule has the form:

$$\tau_{w}\;\frac{dW_{kl}^{ee}}{dt}=r_{l}^{e}\;r_{k}^{e}\;(r_{k}^{e}-\xi_{k});\;\tau_{\xi }\frac{d\xi_{k}}{dt}=-\xi_{k}+(r_{k}^{e}{)}^{2}$$ (3)

where $\tau_{w}$ is the synaptic time scale determining the speed of learning. The BCM threshold $\xi_{k}$ for excitatory neuron *k* is computed by taking the exponential moving average of the neuron’s squared firing rate, with the time constant $\tau_{\xi }$.

We further assumed that each excitatory neuron *k* has limited synaptic resources, which means that the total pre-synaptic connection strength should be preserved throughout the learning process. This constraint can be instantiated biologically through homeostatic processes like synaptic scaling [23], implemented as the weight normalization for each neuron: $\sum_{l}\;W_{kl}^{ee}=w_{ee}(\text{a constant})$. We hypothesized that the synaptic resource constraint serves as a surrogate for the threshold that controls potentiation and depression. This is because the resource constraint induces a selective redistribution: when a postsynaptic neuron fires at a high rate, Hebbian learning strengthens its most correlated inputs, which in turn necessitates a compensatory depression of less correlated inputs to maintain the fixed total weight. To investigate whether the threshold is necessary with the explicit normalization mechanism included, we remove the threshold from the BCM rule and leave the other part of the learning signal the same. The new rule, coupled with weight normalization, is then very similar to the Oja rule [24].

$$\tau_{w}\;\frac{dW_{kl}^{ee}}{dt}=r_{l}^{e}\;(r_{k}^{e}{)}^{2}.$$ (4)

In the following experiments, we refer to this as the “general Hebbian rule” and contrast its outcomes with those of the BCM rule.

### 2.2. Familiarity suppression and tuning curve sharpening in the model

**Fig 2:**
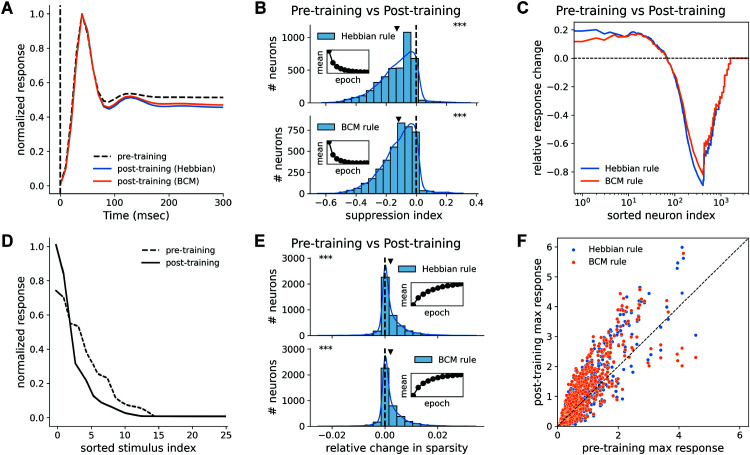
Tuning curve sharpening and familiarity suppression. **(A)** Suppression of the average population response following familiarity training with both BCM and Hebbian rules. The curve represents the average response across all excitatory neurons and all stimuli, normalized according to *r* = *r*/*r*_*max*_, where *r*_*max*_ is the peak response over time. **(B)** Population histograms of SI of individual neurons. Both learning rules result in a statistically significant decrease in the SI. The inset shows the convergence of the distribution’s mean over training epochs. Triangle markers indicate the mean of each distribution. (^***^: *p*<0.001, one-sided t-test against a mean of 0). **(C)** Stimulus-averaged change in population tuning curve. For each stimulus, neuronal responses were sorted in descending order and organized in a log scale. Both rules lead to a sharpening of the population tuning: responses of the most selective neurons are enhanced, while responses of moderately selective neurons are suppressed. Both rules produce comparable profiles. **(D)** Example of a single neuron’s tuning curve across 25 stimuli before (blue) and after (orange) familiarity training. The tuning curve sharpens, characterized by an enhanced response to the most preferred stimulus and suppressed responses to non-preferred stimuli. **(E)** Population histograms of the relative change in the lifetime sparsity. Both learning rules produce a statistically significant increase in lifetime sparsity, indicating that neuronal tuning becomes more selective. The inset shows the convergence of the distribution’s mean over training epochs. (^***^ : *p*<0.001, one-sided t-test against a mean of 0). **(F)** Scatter plot of maximum values of tuning curves pre- and post-training. Both rules result in an increase in peak response in the responsive neuron.

Our model successfully reproduced familiarity suppression under both the general Hebbian and BCM learning rules. To achieve this, the network was trained for 80 epochs on a set of 25 natural images from the CIFAR-100 dataset, with each image presented for 300 simulation steps per epoch. Fig 2A displays the peri-stimulus firing rate, averaged across all excitatory neurons and stimuli, before and after training. Following stimulus onset, the network exhibits a characteristic dynamic: a sharp transient peak followed by a decay as surround suppression takes effect, finally settling into a steady state. Critically, after training, the average steady-state population response to these familiar stimuli is markedly suppressed under both the Hebbian (blue) and BCM (orange) rules compared to the pre-training response (dashed line). To confirm this suppression at the single neuron level, we analyzed the stimulus-average suppression index (abbreviated SI, the relative change of each neuron’s stimulus-averaged firing rate measured by (post - pre) / (post + pre)) during the steady-state period. Fig 2B shows that both learning rules result in a comparable proportion of neurons exhibiting a decrease in SI.

In addition to population-wide suppression, the network exhibited significant tuning curve sharpening for familiar stimuli. Fig 2D shows a representative sharpened tuning curve, where the neuron’s response to its most preferred stimulus is enhanced while responses to other stimuli are suppressed. To quantify this effect across the population, we measured both lifetime sparsity (intuitively, the area-under-curve in Fig 2D; see Sect 4.3) and peak firing rate. For both the Hebbian and BCM rules, familiarity training induced a significant positive shift in the lifetime sparsity across the population (Fig 2E). Meanwhile, the responsive neurons (which have a high affinity to the 25 images stimuli) showed a marked increase in the peak response (Fig 2F). The increased lifetime sparsity and peak response together quantitatively verified the characteristic of the sharpened tuning curve (Fig 2D) across the neuron population.

These results suggest the formation of specialized local circuits, or cell assemblies, dedicated to familiar stimuli. The excitatory connections of the neurons in the assembly boost one another’s responses, amplifying their responses to the preferred stimulus, resulting in an increase in peak response. This amplified activity, in turn, would drive stronger inhibition of surrounding neurons via surround suppression and divisive normalization, producing the net decrease in the average population response. The increase in the lifetime sparsity is then a synergistic effect of selective amplification and feedback inhibition. This proposed mechanism is directly supported by the stimulus-averaged population tuning curve (Fig 2C). In this plot, the population responses of the neurons were sorted according to rank order for each stimulus, and then averaged across stimuli. It shows that only the most selective neurons are enhanced post-training, while all others are suppressed. The fact that both learning rules produce these comparable changes across all key statistics—population suppression, lifetime sparsity, and population tuning curve—demonstrates that both are valid mechanisms for generating the full suite of familiarity effects.

### 2.3. Relating familiarity-trained recurrent circuit and manifold transform

In this section and the following, we investigate the computational consequences of forming a recurrent circuit in the early visual area through familiarity training on the manifold geometry of neural response space [25,26] for representing a set of global images. Inspired by the recently proposed sparse manifold transform framework [13,27], we propose that the recurrent circuit learned by familiar training performs a manifold transform that maps the input representation manifold to a representation manifold in which perceptually related or similar images become proximal in this manifold, thus better reflecting the geometry of the image manifold and facilitating the computation of invariant representations of visual concepts downstream.

#### The objective of manifold transform.

**Fig 3:**
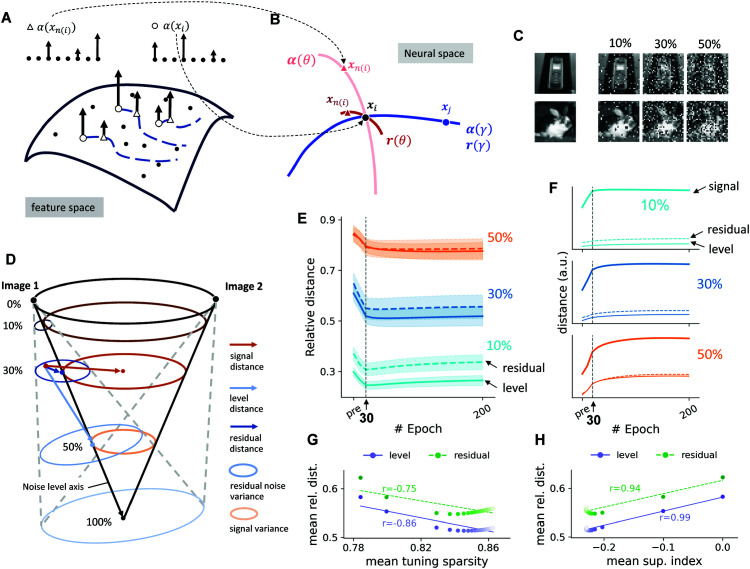
Manifold transformation in the familiarity association experiment. **(A)** Conceptual illustration of a visual feature manifold. Consider a smooth surface of local visual features on which a sparse coding dictionary provides discrete samples (ℳ) [13]. Nuisance transformations of a visual stimulus, such as noise or changing viewpoints, correspond to flows along this manifold (blue dotted lines). The circles and triangles represent distinct sets of activated dictionary elements for two perceptually similar stimuli generated by the nuisance transformation (*x*_*i*_ and *x*_(*n*(*i*))_), with arrows indicating response amplitudes. Because the dictionary is unordered, a smooth manifold flow can result in dissimilar sparse code activations (top). **(B)** Schematic of the manifold transform performed by the learned circuit. The model considers two types of relationships: a ’concept manifold’ (blue curve) representing the neural codes of distinct stimuli such as a target image *x*_*i*_ (black dot) and a dissimilar stimulus *x*_*j*_ (blue dot); and a ’variants manifold’ (red curve) representing the neural codes of variations of the same concept, such as *x*_*i*_ and a related similar stimulus *x*_*n*(*i*)_ (red dot). The learned circuit encoder, $\phi_{𝐖}$, maps the sparse codes to a new representation, **r**. This transformation compresses the variants manifold, reducing the distance between *x*_*i*_ and *x*_*n*(*i*)_ to better reflect their geometric relationship. For visual simplicity, the concept manifold is depicted as unchanged by the encoder. **(C)** Example images from the stimulus set (CIFAR100, publicly available here, also see [21]), corrupted with 10%, 30%, and 50% salt-and-pepper occlusion noise. **(D)** Schematic of the neural manifold geometry in the familiarity association experiment. The orange rings represent the signal variance for stimuli at different noise levels, forming a “signal cone.” Each target image and its corrupted samples would form an opposite “noise cone”. The blue rings represent the noise variances inside the noise cone at varying noise levels. From a specific noise sample (the red dot), the level distance is marked by the light blue arrow, the residual distance is marked by the deep blue arrow, and the signal distance is marked by the orange arrow. **(E)** Across noise levels, $R^{lev}$ and $R^{res}$ over the first 200 epochs show a two-phase trajectory: a minimum at epoch 30 (dashed line), and an overall decrease. This indicates a compression of the neural manifold in both level and residual directions. Ribbons represent the standard deviation across different target images. **(F)** $D^{sig}$, $D^{lev}$ and $D^{res}$ over first 200 epoch at all noise levels. The net decrease in both relative distances is primarily due to the larger increase in $D^{sig}$. **(G, H)** The relative distances exhibit a reverse correlation with neuronal tuning selectivity and the magnitude of SI. The darkness of the scatter indicates the number of epochs, with deeper colors corresponding to earlier epochs. The solid lines represent the fitted regression lines, with the corresponding Pearson correlation coefficient noted aside.

Research in manifold learning has demonstrated that primary sensory areas such as V1 cannot represent the geometric relations between semantically or perceptually similar images, because their neurons are selective to local features, such as those in our model’s sparse code dictionary [11,13,28]. A small nuisance transformation of a global image, e.g., changes in view of an object, or adding occlusion noises can drastically change the representation in the sparse population codes [13,27], inducing a large distance between the two perceptually similar images in the neural space (Fig 3A, 3B). The manifold transform aims to find a representation whose geometry is more consistent with the perceptual similarity structure of the images.

For each image concept *i* and nuisance condition *θ*, we write the stimulus as $x_{i,\theta }\;=\;{𝐬}_{i}\;+\;\eta_{\theta }$; thus, variants share a concept-specific signal (${𝐬}_{i}$) and differ by nuisance-specific residuals ($\eta_{\theta }$). A sparse-coding front end produces α(x), and the recurrent circuit acts as an encoder $\phi_{𝐖}$ (parameterized by $𝐖\;:={𝐖}^{ee}$), mapping α(x) to a steady-state representation $𝐫=\phi_{𝐖}(\alpha (x))$ that preserves the perceptual-similarity structure in the stimuli. Given this decomposition, we define the induced manifolds in either representation space (α and **r**):


$${ℳ}_{\theta }^{\alpha }(i)\;=\;\{\;\alpha (x_{i,\theta })\;:\;\theta \in \Theta_{i}\;\},\;{ℳ}_{\gamma }^{\alpha }(\theta )\;=\;\{\;\alpha (x_{i,\theta })\;:\;j\in \Gamma_{\theta }\;\},$$

$${ℳ}_{\theta }^{r}(i)\;=\;\{\;𝐫(x_{i,\theta })\;:\;\theta \in \Theta_{i}\;\},\;{ℳ}_{\gamma }^{r}(\theta )\;=\;\{\;𝐫(x_{i,\theta })\;:\;j\in \Gamma_{\theta }\;\}.$$

For brevity, we refer to ${ℳ}_{\theta }$ as the *θ* manifold (variants manifold) and to ${ℳ}_{\gamma }$ as the *γ* manifold (concept manifold).

The learned representation **r** needs to satisfy the following property compared to the sparse code α: the stimulus *x*_*i*_ should be closer to similar stimuli *x*_*n*(*i*)_ that are in the same variants manifold, relative to dissimilar stimuli *x*_*j*_ that are in other variants manifolds, i.e., the manifold transform should compress all the variants manifolds relative to the concept manifold (Fig 3B), minimizing the effects of nuisance transformations. Such a manifold transform can be defined as a learning objective function that acts to minimize the distance between the representations of stimuli within the variants manifold of each image concept while maintaining the distinction between the representations of the different image concepts:

$$ℒ(𝐖)=\sum_{i}\;\frac{{𝔼}_{k\in n(i)}[\;\|\;{𝐫}_{𝐖}(x_{i})-{𝐫}_{𝐖}(x_{k})\;{\|}^{2}\;]}{{𝔼}_{j}[\;\|\;{𝐫}_{𝐖}(x_{i})-{𝐫}_{𝐖}(x_{j})\;{\|}^{2}\;]};\;\;{𝐫}_{𝐖}(x_{i})=\phi_{𝐖}(\alpha (x_{i})),$$ (5)

where *x*_*i*_ and *x*_*k*_ are the similar stimuli, whereas *x*_*i*_ and *x*_*j*_ are dissimilar stimuli across the concept manifold, and $\phi_{𝐖}$ is the manifold transform, in our case, implemented by the recurrent circuit.

#### Experiment to link manifold transform and familiarity training.

To establish the relationship between familiarity training and the manifold transform, we designed the following simulation experiment to demonstrate that the objective function above indeed decreases during familiarity training. In this experiment, we trained the network with five global image concepts. For each concept, we will also train its variant images, characterized by different degrees of salt and pepper noise occlusions. Fig 3C shows two example visual concepts, i.e., noiseless global target images, as well as examples of these two concepts corrupted by 10%, 30%, and 50% of noise. Corruption of target image *l* with noise level *n* results in a conditional distribution of stimuli p(x|n,l). We draw 10 noisy samples denoted by $x_{n,l}^{k},\;k=1,\ldots ,10$ from each conditional distribution for each target image at each noise level.

This design allows us to explore the *θ* manifold in two dimensions, across noise level and within each noise level. Fig 3D illustrates the geometric relationship between the two example stimuli and their variations across noise levels and within noise levels. The *θ* manifold for each stimulus is a cone, with the axis of the cone (noise-to-signal axis) spanning the noise level, and the cross-section of the cone representing the distribution of samples within each noise level, referred to as residuals (S1 Fig, panel B). The *γ* manifold is an opposite cone, reflecting the reduced signal contents in the corrupted images as the noise level increases (S1 Fig, panel C). For 100% noise, all the “images” will converge to the same cloud.

The manifold transform predicts that familiarity training compresses the variants manifold relative to the concept manifold. For a sample *k* at noise level *n* and target *l*, with steady-state response 𝐫(x), we define: *level distance* (adjacent lower noise, same target):


$$D_{n,l,k}^{lev}={𝔼}_{k^{\prime }}\;\left[{\left\|𝐫\;\left(x_{n,l}^{k}\right)-𝐫\;\left(x_{n-1,l}^{k^{\prime }}\right)\right\|}^{2}\right]\;\text{(light-blue arrow in Fig\textasciitilde{}4D)};$$

*residual distance* (same noise and target):


$$D_{n,l,k}^{res}={𝔼}_{k^{\prime }}\;\left[{\left\|𝐫\;\left(x_{n,l}^{k}\right)-𝐫\;\left(x_{n,l}^{k^{\prime }}\right)\right\|}^{2}\right]\;\text{(dark-blue arrow)};$$

and *signal distance* (other targets at the same noise level):


$$D_{n,k}^{sig}={𝔼}_{\;l^{\prime }\neq l,\;k^{\prime }}\;\left[{\left\|𝐫\;\left(x_{n,l}^{k}\right)-𝐫\;\left(x_{n,l^{\prime }}^{k^{\prime }}\right)\right\|}^{2}\right]\;\text{(orange arrow)}.$$

We then form relative (level and residual) distances via normalizing by the signal distance:


$$R_{n,l,k}^{lev}=\frac{D_{n,l,k}^{lev}}{D_{n,k}^{sig}},\;R_{n,l,k}^{res}=\frac{D_{n,l,k}^{res}}{D_{n,k}^{sig}}.$$

Relative compression of the variants manifold corresponds to decreases in $R^{lev}$ and $R^{res}$ during familiarity training. These ratios are specific instantiations of the objective in Eq 5, obtained by evaluating its numerator along across-level and within-level nuisance dimensions, respectively.

In total, there are 155 different stimuli we used in the familiarity training. Each target image was trained 30 times, while each of its 30 different variants was trained once in each epoch. The network is trained for 350 epochs, where each input image was presented once. The set of stimuli was shuffled and presented in random sequences. Our Analyses were centered on the general Hebbian model; BCM metrics are presented in the Supplementary (Fig A2B and Sect B in S1 Text).

#### Results of the simulated experiment.

After each epoch of training, we probed the population activity and analyzed the neural representation manifold formed by fixed points. The evolution of the population activity in response to stimuli of different noise levels forms distinct trajectories in the neural representation space, which start at the same initial resting state of the network, then diverge and settle into different fixed points corresponding to the distinct input images. Fixed points of different noise levels are organized along certain directions in the neural representation space, forming a signal-to-noise axis along which the noise level increases gradually (S1 Fig, panel A).

We then computed $R^{lev}$ and the $R^{res}$. We observed that both relative distances decreased in the early training stages, followed by a modest, gradual rebound in later training epochs, and ultimately exhibited a net decrease. Additionally, the reduction was more pronounced at higher noise levels (Fig 3E). These results confirm that familiarity-driven training effectively compresses the variants manifold relative to the concept manifold. We found that the observed reductions in both *R* are primarily due to the expansion of the concept manifold. There is a larger increase in $D^{sig}$ compared to $D^{lev}$ and $D^{res}$ (Fig 3F), indicating that the mutual excitation of the neurons that encodes the familiar stimulus context have led to a selective amplification of the concept-specific signal component [29–31]. Interestingly, for both networks, the relative compression of the variant manifolds was strongly correlated with the observed familiarity effects, specifically the increase in tuning selectivity, and an increase in SI (Fig 3G, 3H). This suggests that the compression of the neural representation and the familiarity effects observed in neurons are tightly linked, potentially through a common underlying driver or a direct causal relationship.

We further assessed the impact of the manifold transform in the primary visual cortex on the higher-level visual area. We trained individual neurons to represent specific familiar concepts encoded in the trained circuit with a competitive learning rule that partitioned the representation space into concept clusters, each associated with a concept neuron (see Sect D in S1 Text). We found that familiarity training significantly increased the concept selectivity of these downstream concept neurons (Fig A4 in S1 Text). In addition, we found that training the network with the clean target images alone, without training the noise samples, is sufficient to produce essentially the same effect (Fig A2A in S1 Text). This is consistent with the observation that the relative compression of the variants manifold is primarily due to the expansion of the concept manifold. Together, these results demonstrate that familiarity training induces a change in representation consistent with the proposed manifold transform, compressing the variants manifolds relative to the concept manifolds.

### 2.4. Linear system analysis to dissect the mechanisms of manifold transform in the recurrent circuit

We have shown that familiarity training compressed the noise variant manifold primarily by increasing the signal distance $D^{sig}$ (Fig 3E, 3F), suggesting that the familiarity training modulates gain of the recurrent circuit in a selective manner. In this section, we will first establish when the manifold transform in the recurrent circuit is locally linear around each attractor, and then we will use linear system analysis to reveal how mechanistically the recurrent circuit expands the concept manifold relative to the noise variant manifold through familiarity training.

#### A local linear strategy for manifold transform in recurrent circuit.

**Fig 4:**
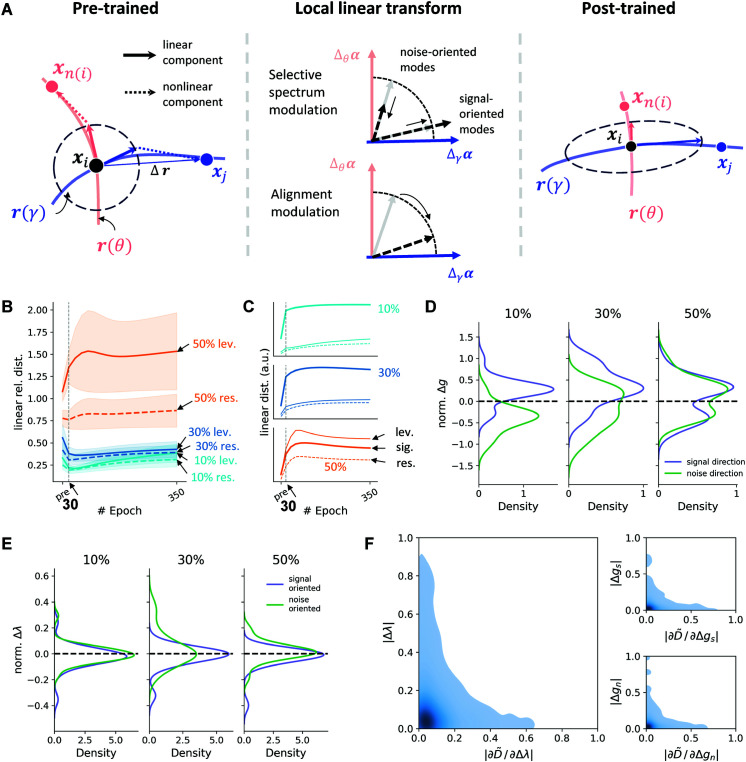
A Locally Linear Dynamic Strategy for Manifold Learning. **(A)** This schematic illustrates how a recurrent circuit can perform a manifold transform. On the left, the red and blue curves represent the variants *θ* manifold and the concept *γ* manifold, respectively. Similar to Fig 3B, the black dot is the representation of stimulus *x*_*i*_, and the red dot is the positive sample, the blue dot is the negative sample. The displacement between two attractors (Δ𝐫) on the manifold decomposes into locally linear (within the vicinity of the attractor, denoted by the dashed circle) and globally nonlinear components, and the network can reshape the manifold by modulating either. The local linear transform is the strategy that manipulates the local linear component to drive global geometric changes. The objective of the local linear strategy is to anisotropically adjust network’s recurrent gain, thereby stretching the signal geometry while compressing the noise (pre-training vs post-training). This can be achieved via two possible mechanisms (middle): selective spectrum modulation (top), which increases the spectrum of signal-oriented modes and decreases the spectrum of noise-oriented modes, or alignment modulation (bottom), which rotates the modes to be more signal-oriented. The blue and red arrows represent signal and noise directions in the input α space ($\Delta_{\gamma }\alpha$ and $\Delta_{\theta }\alpha$, respectively). The grey, solid arrow represents pre-training collective modes, and the black, dashed arrow represents post-training collective modes. **(B)** Evolution of the linearized $R^{lev}$ and the $R^{res}$ across three different noise levels. For 10–30% noise, *R* shows an early drop (maximal linear compression, epoch 30) that parallels the full metrics; at low noise, *R* rebounds in the late training stage while the full metric remains reduced, indicating additional nonlinear contributions. At 50% noise, *R* increases, marking a regime not well captured by local linearization. **(C)** Corresponding evolution of linearized $D^{sig}$, $D^{lev}$ and $D^{res}$ used to calculate *R* in panel B, which also mirrors full distances at low-to-mid noise levels in the early stage (compare to Fig 3F). **D–F** *are shown at epoch 30, the time of maximal linear compression for 10–30% noise to isolate the locally linear mechanism.* **(D)** Density distributions of the normalized change in modes’ alignment (Δg) to signal versus noise direction. For 10-30% noise, where the Hebbian network employs a local linear transform, learning selectively increases signal direction alignment (blue) while simultaneously decreasing noise direction alignment (orange). The noise alignment here represents the average of the level and residual alignments. **(E)** Density distributions of the normalized change in the collective mode spectrum (Δλ) for signal-oriented modes versus noise-oriented modes. In contrast to alignment modulation, the change in the spectrum is largely non-selective. Δλ exhibits no significant change for both signal-oriented (blue) and noise-oriented (orange) modes. The signal-oriented modes are those that align more with the signal direction in the input α space pre-training, and similarly for the noise-oriented modes. The noise alignment here represents the average of the level and residual alignments. **(F)** Large plot on the left: Joint density plot showing the relationship between the normalized sensitivity magnitude ($\left|\partial \tilde{D}/\partial \Delta \lambda \right|$) and the normalized change magnitude (|Δλ|) for the spectrum. The two small plots on the right show similar relations for signal and noise alignment ($\Delta g_{\text{s}}$ and $\Delta g_{\text{n}}$). The sensitivity quantifies the contribution of the change in mode alignment or spectrum to the linear relative distance. The three density plots reveal a consistent inverse relationship: learning primarily modifies modes that were initially insensitive (low sensitivity, high change, top-left cluster), while leaving highly sensitive modes largely unchanged (high sensitivity, low change, bottom-right cluster).

As shown in Fig 4A, the displacement from one image *x*_*i*_ to another image *x*_*j*_ on the representation manifold (denoted by Δ𝐫) can be decomposed into a linear component and a nonlinear component. The linear component dominates in the vicinity of the point *x*_*i*_, indicated by the dotted circle. To reshape the manifold’s overall geometry, the network can modulate *either* of these components. Local linear transform is a strategy networks use to manipulate the local linear component to drive global geometric changes. This approach is analogous to the “fit locally, think globally” principle, which is foundational to many manifold learning algorithms [11,13,14]. According to this principle, the global structure of a manifold can be accurately reconstructed by preserving local neighborhood relationships, ensuring nearby points on the original manifold remain neighbors in the new representation.

We considered how recurrent dynamics would affect the norm of the local linear component. A key property of nonlinear recurrent dynamics is that, near an attractor, the network’s input-output properties can be accurately captured by a linear, first-order approximation defined by a recurrent gain matrix *M*. The influence of this recurrent circuitry can be decomposed along a set of *recurrent gain modes* (see Sect 4.5 for details). These modes represent specific patterns of neural activity that are selectively amplified or attenuated by the network, with the degree of modulation for each mode determined by its corresponding eigenvalue. Crucially, the norm of the linear component is not determined by these gain modes in isolation, but by their interactions. These interactions give rise to a set of emergent *collective modes* (see Sect 4.5 for details), which capture the interaction pattern of the recurrent gain modes. Specifically, the collective modes arise from the non-orthogonal geometry of the gain modes and provide an orthogonal basis that diagonalizes their interactions. Each collective mode thus represents a pattern of activity whose eigenvalue (spectrum) quantifies the net amplification arising from this constructive and destructive interference. The expected squared norm, $E[\Delta {𝐫}^{⊤}\Delta 𝐫]$, can be expressed in terms of these collective modes as:

$$\langle \Delta {𝐫}^{⊤}\Delta 𝐫\rangle =\sum_{k=1}^{N}\;\underset{\text{spectrum}}{\underbrace{\lambda_{k}}}\;\underset{\text{alignment}}{\underbrace{\varphi_{k}^{⊤}\;\langle \Delta \alpha \Delta \alpha^{⊤}\rangle \;\varphi_{k}}}.$$ (6)

Each term in the sum is the product of two factors: first, the “spectrum” term $\lambda_{k}$, which is the eigenvalue of the *k*-th collective mode, representing its amplification gain, second the “alignment” term, which measures how well the direction of input variations, $\Delta \alpha^{⊤}$, align with the *k*-th mode’s effective input filter $\varphi_{k}$ (see Sect 4.5).

We define the signal directions as the vectors of variations on the *γ* manifold (blue curve in Fig 4A, denoted by $\Delta_{\gamma }$), and the noise directions as the vectors of variations (both across noise levels and residual noise, denoted by $\Delta_{\theta }$) on the *θ* manifold (red curve in Fig 4A). Here, we use “noise” as a general term for nuisance variables or irrelevant transforms, though occlusion noise will be used as a type of nuisance variable in our simulation study. The objective of the local linear transform is to adjust the recurrent gain of the linearized network anisotropically: amplifying it along the signal direction while suppressing it along the noise direction. This effectively stretches the local geometry along the relevant signal direction and compresses it along the irrelevant noise direction (Fig 4A, Pre-trained and Post-trained). This can be implemented via two possible mechanisms: (1) The selective spectrum modulation (Fig 4A, middle top), which creates a spectral gap by amplifying the spectrum of signal-oriented modes (modes that align more with the signal direction in the input α space ($\Delta_{\gamma }\alpha$) in the pre-training stage) while suppressing the spectrum of noise-oriented modes (modes that align more with the noise direction in the input α space ($\Delta_{\theta }\alpha$) in the pre-training stage), without changing the direction of modes. (2) The alignment modulation (Fig 4A, middle bottom), which rotates the principal collective modes to align more closely with the $\Delta_{\gamma }\alpha$, without selectively modulating the spectrum based on the mode direction pre-training.

#### Recurrent networks employ local linear transform at low-to-mid noise levels in the early stage.

To investigate whether the familiarity-trained network employed the local linear transform, we numerically linearized the network around each attractor and computed $R^{lev}$ and the $R^{res}$ using the first-order approximation. As shown in Fig 4B, for 10–30% noise, the early decrease and the timing of the minimum in the full relative distances are captured by the linear model, indicating that the initial compression of the variants manifold is predominantly linear. Consistent with this, the linearized $D^{sig}$, $D^{lev}$ and $D^{res}$ track their full counterparts in this phase: both increase, with the signal component rising more steeply than the noise component, and matched elbow points at epoch 30, resulting in the early drop in the relative distances (Fig 4C and Fig 3F). After this locally linear phase, the linear metric partially rebounds with a net increase at 10% by the end; thus, the net decrease at 10% in the full relative distances reflects additional nonlinear contributions. By contrast, at 50% noise, the linear model predicts an increase in relative distance, indicating that input variation has moved the system beyond the local-linear regime, where global nonlinear effects dominate the manifold transform (Fig 4B—4C). Together, these findings revealed a two-phase change at the low-to-mid noise level: locally linear compression followed by nonlinear consolidation, and an overall nonlinear, global compression at the high noise level.

#### The local linear transform is implemented by alignment modulation.

As shown in Fig 3E, the recurrent circuit compresses the variants manifold relative to the concept manifold, reaching a minimum around epoch 30 and then roughly plateauing. This early trajectory is captured by the linear approximation (Fig 4B–4C). After epoch 30, the linear model begins to lose compression for certain noise levels, diverging from the full network (Fig 3E), indicating emerging nonlinear effects for the maintenance of the relative compression. These later nonlinearities appear to have a secondary impact on the relative manifold compression, as the most significant change in relative distance appears before 30 epochs (Fig 3E). Accordingly, we focus the mechanistic analysis on the locally linear phase (≤ epoch 30), which isolates the primary driver of the manifold transform.

We now tested which of the two mechanisms (alignment vs spectrum modulation) drives the local linear transform at low–mid noise by focusing on epoch 30. We found that the data are not consistent with the selective spectrum modulation hypothesis. Fig 4E shows that the change in the collective mode spectrum (Δλ) is largely non-selective. The distributions of Δλ are almost identical whether the modes are primarily oriented towards the signal direction or to the noise directions. In contrast, the results support the alignment modulation strategy. Within the effective linear regime (10% and 30% noise), the distributions of normalized alignment change (Δg) exhibit an increase in the alignment with the signal direction (blue distributions, Fig 4D) and a decrease in the alignment with the noise direction (orange distributions, Fig 4D).

#### The local linear transform primarily recruits low-sensitivity modes.

Finally, we investigated how the magnitude of alignment modulation and spectrum strengthening of each mode are related to their contribution to the linear relative distance (*R*). With $\tilde{D}=\Delta D/D_{\text{pre}}$, the fractional change in the individual noise or signal distance, our linear analysis reveals R↓ when ${\tilde{D}}_{\text{sig}}>{\tilde{D}}_{\text{noise}}$ (see Sect 4.7). We thereby defining the sensitivity of the linear relative distance with respect to the change in model spectrum or mode alignment as $\partial \tilde{D}\;/\;\partial \Delta \lambda$ or $\partial \tilde{D}\;/\;\partial \Delta g$, which are determined by the initial values in the alignment *g* or the spectrum *λ* in the pre-trained phase, respectively (see Sect 4.7). An efficient learning strategy is expected to primarily modify the most sensitive modes, allowing for a stronger change in compression with the same amount of change in the mode spectrum and alignment.

However, our analysis reveals that the network employs the opposite strategy. Fig 4F shows the joint density of each mode’s sensitivity magnitude against the magnitude of its change at epoch 30, the time of maximal linear compression for 10–30%. The distribution is highly non-uniform and dominated by three distinct features. The vast majority of modes are concentrated in a dense peak near the origin, corresponding to null modes with both low pre-training sensitivity and minimal change during learning. From this central mass, the distribution extends into two sparse tails. The vertical tail represents a population of initially weak modes that are subject to large modifications. This group constitutes the network’s plasticity budget: modes that are recruited during familiarity learning. Conversely, the horizontal tail represents the network’s stable, high-sensitivity core modes, which are left untouched during training.

While this strategy may seem less efficient from an optimization perspective, it may represent a more robust and stable solution: modifying dominant, high-sensitivity modes could risk destabilizing the network’s computational dynamics. By preserving these modes, the network would also be protecting its foundational memories from being overwritten, thus mitigating catastrophic forgetting. In this view, the network utilizes its large pool of weak, low-sensitivity modes as a flexible “plasticity budget,” allowing it to integrate new computational functions.

## 3. Discussion

Neurophysiological observations indicate that familiarity effects depend on contextual information beyond a neuron’s classical receptive field. Furthermore, these effects appear earliest in V1, then in V2, and subsequently in IT, suggesting the emergence of horizontal recurrent circuits within each visual area along the visual hierarchy. In this work, we demonstrate that the suppression of population-averaged responses to familiar images in V1 and V2, as reported by Huang et al. [8], can be accounted for by the formation of local recurrent circuits linking excitatory neurons across hypercolumns in a canonical V1 circuit. These circuits rely on well-established mechanisms, including Hebbian learning, within-hypercolumn normalization, and iso-feature-channel normalization (generalized from iso-orientation suppression) across hypercolumns.

Our model shows that the formation of such local recurrent circuits can enhance the responses of neurons involved in encoding a specific image context, while suppressing the activity of uninvolved neurons. This leads to a sparser neural code and sharper tuning, effectively enhancing both single-neuron and population-level selectivities for the familiar stimuli. While Huang et al. [8] did not find statistically significant evidence in support of tuning curve sharpening when training and testing only 25 familiar images, our model predicts that sharpening should emerge with a sufficiently large set of recorded neurons. Indeed, tuning sharpening for familiar stimuli has been observed in the inferotemporal cortex [7]. Our preliminary experiments on one monkey also found that V2 neurons exhibit sharpening when 200 stimuli were tested.

Lim and colleagues [22] showed that the plasticity rule underlying the sharpening effect of familiarity learning to resemble a BCM-like learning rule, with LTD (long-term depression or decrease in synaptic weight) when the firing rate of the postsynaptic neuron in response to a stimulus is below a certain threshold, and LTP (long-term potentiation or gain in synaptic weight) when the firing rate is above the threshold [22]. Our simulation using networks utilizing BCM-learning rule successfully reproduced the familiarity suppression and representation sparsification (Fig 2). However, we found that similar effects (i.e. suppression and sparsification, as well as manifold transform) can also be achieved using a Hebbian learning rule without an explicit firing-rate threshold, when it is combined with a weight normalization mechanism as in Oja’s rule (Fig 2). In this case, LTD is mediated effectively by synaptic scaling, obviating the need for a BCM-type threshold mechanism.

The recurrent circuit formed through familiarity learning functions as a local Hopfield network that encodes specific episodic image memories even in early visual areas. Why might the early visual cortex be involved in encoding global image memories when IT, where neurons have larger receptive fields for encoding entire object or scene representations, is already encoding them? One possibility is that early encoding of global image structures, such as faces [32–34], can support faster recognition and decision-making. Another possibility is that encoding global image structures by dynamically linking elementary elements at early visual areas is a form of compositional learning that gives flexibility and versatility in object representation [35–38]. Here, we propose a third idea that the recurrent circuits encoding global images implement a manifold transform to compress irrelevant dimensions, thereby helping downstream neurons in higher-level visual areas achieve invariant representations explicitly. Specifically, we proposed that recurrent cortical circuits perform a locally linear but globally nonlinear transformation of neural manifolds, and that familiarity training induces structured modifications to this transformation. Using network simulations, we showed that familiarity training indeed optimizes an objective function associated with manifold transformation: dimensions corresponding to nuisance variation or noise are selectively compressed relative to those encoding meaningful signals. Specifically, familiarity training was found to reduce distances along noise directions while preserving or enhancing distances along stimulus-relevant dimensions near each attractor—behavior consistent with the goals of manifold transformation, leading to more robust and discriminative representations of global image concepts.

The recurrent circuit learned through familiarity training induces sparsification of neural tuning, leading to reduced population-level activity, as a form of efficient coding. We found that the optimization of the manifold transform objective was positively correlated with both increased selectivity and population suppression for familiar images. This implies that sparsification co-occurs with the rotation of collective modes around each attractor, jointly contributing to the observed manifold transform. One possible explanation is that aligning the collective modes with signal dimensions and orthogonalizing them to specific irrelevant (e.g., occlusion noise level *θ*) directions reduces representational interference between concepts, thus promoting sparsity [39]. However, the precise relationship between sparsification and mode rotation remains an open question, and further research is needed to fully elucidate their interaction during Hebbian-based familiarity learning.

From a classical perspective, neurons in higher visual areas such as the inferotemporal cortex (IT) achieve invariance to transformations including translation, rotation, scale, and changes in viewpoint. This invariance emerges progressively along the visual hierarchy, with intermediate areas exhibiting intermediate degrees of invariance—striking a balance between object specificity and generalization. Classical neural network models, such as the Neocognitron [40] and modern convolutional neural networks (CNNs) [41], realize this process through purely feedforward architectures. In contrast, we propose that recurrent circuits play a critical role, perhaps complementary to feedforward connections, in achieving these invariances via manifold transformations. Each visual area contributes to invariance at a spatial scale that corresponds to the size of its neurons’ receptive fields [35,42]. The invariance generated by recurrent processing is thus locally appropriate to the scale of each area, while more global invariance emerges at higher-level visual areas through the hierarchical structure. Neurons in each visual area read out and build upon the locally invariant population representations from preceding areas (Fig A4 and Sect D in S1 Text). These inputs, combined with the area’s own recurrent dynamics and potentially feedforward signals, allow individual neurons to encode more explicit and robust invariance in their tuning properties. For instance, population-level representations in V1—shaped by manifold transforms via recurrent circuits—can support the development of invariant responses in individual neurons in V2. In turn, the recurrent circuitry in V2 can facilitate the emergence of more complex invariant representations in V4.

Manifold-learning methods (e.g., [12,13]) first construct a local-neighbor graph that encodes approximate linear structure and then compute a single global embedding consistent with that graph. These methods implicitly assume dense, roughly uniform sampling; with sparse or anisotropic data, local-linear reconstruction and the graph itself can become unreliable. In real visual tasks such as our familiarity association experiment, the transform must be learned online from a stream of stimuli: the animal does not have access to a complete, uniformly sampled dataset a priori. This violates the uniform-density assumption and motivates a locally linear, globally nonlinear strategy implemented by recurrent dynamics. On the one hand, due to the nature of recurrent dynamics near attractor states, the system’s behavior becomes approximately linear in a local neighborhood. Within this regime, a neural manifold can be modified by rotating the collective modes of recurrent gain relative to task-relevant signals or irrelevant variation. Our network analysis results suggest that, for spatially close stimuli, such as corrupted images with low-to-mid noise levels (10% or 30%), familiarity learning leads to such rotations, aligning collective modes more closely with signal dimensions while orthogonalizing them to noise or irrelevant variability. Interestingly, we found that the network selectively favors modifying those modes that have weak initial strengths, perhaps as a strategy to preserve computational dynamics and avoid disrupting existing associative memories. On the other hand, under extreme distortions, such as 50% occlusion, the stimuli become farther apart, violating the locality assumption. In such cases, the underlying manifold objective may still be achieved through nonlinear dynamics. Thus, the trained recurrent computation facilitates flexible and stable manifold transforms centered around familiar concepts.

Several limitations to this study warrant future investigation. First, the architecture of our model does not fully replicate the biological complexity of cortical circuits, such as diverse neuron types and detailed connectivity patterns. Instead, we adopted a simplified canonical circuit model incorporating standard features of macaque V1—such as surround excitation/inhibition and normalization—to examine the core mechanisms that may generalize to other areas like V2, V4, and IT. Future work should explore how incorporating more biologically realistic circuitry could affect the model’s explanatory power for familiarity effects and its ability to implement manifold transformations.

A second open question concerns the broader applicability of the proposed manifold transformation framework. While our model successfully handles occlusion noise, its effectiveness across other continuous transformations—such as rotation, translation, contrast variation, spatial frequency modulation, or dynamic temporal stimuli—remains to be determined. It is possible that the Hebbian rule is specifically suitable for the image statistics of the occlusion noise. Thus, a more general learning rule is required to extract and selectively enhance the gain along signal directions that correspond to the image content, among the noise directions corresponding to nuisance variables of different types. These generalizations may also require modifications at the circuit level, but we argue that the principles uncovered here—local recurrence, Hebbian learning, and normalization—form a canonical computational motif that can support familiarity learning across the visual hierarchy. But the relative contribution of recurrent connections and feedforward connections and their synergistic interaction in creating an increasing degree of invariance remains to be characterized.

Another open question is that the familiarity training used here is unsupervised: clean targets and their noisy variants are randomly interleaved, removing the temporal structure that could act as an external supervisory signal. A key direction for future research is to investigate how recurrent circuits can be extended to process video data and to determine how temporal associations between images might be leveraged as a form of supervision to guide manifold learning, like in the slow feature analysis [28].

In closing, manifold transforms in modern machine learning (e.g., [11–13,27]) are powerful: they first build a neighborhood graph from the full dataset and then obtain a single, global low-dimensional embedding by solving an eigenproblem. In contrast, this paper shows that a recurrent circuit with familiarity training can implement a locally linear yet globally nonlinear manifold transform, offering a biologically plausible alternative strategy adopted by the brain.

## 4. Methods

### 4.1. Feedforward response

The feedforward response to an input image was computed using a set of *N*_*d*_ = 64 convolutional filters. These filters were pre-trained using a convolutional sparse coding algorithm [18], a method known to yield efficient codes and receptive fields similar to those in the primary visual cortex [43]. The algorithm jointly optimizes a dictionary of filters, *D*_*i*_, and the corresponding sparse activations, $\alpha_{i}$, by minimizing the following objective over a set of training image patches, *I*_*m*_:

$$\min_{\alpha }\|\sum_{j=1}^{k}D_{j}\;*\;\alpha_{j}-I_{m}{\|}^{2},\;\text{s.t. }\|\alpha {\|}_{0}<q$$ (7)

This objective function minimizes the reconstruction error subject to an L1 sparsity penalty. For all subsequent familiarity experiments, these pre-trained filters were held fixed. To compute the feedforward response for a given input image, the image was convolved with the 64 learned filters (size 9×9), using a stride of 3 and no padding, which resulted in 8×8 feature maps for each filter. We set the size of the network accordingly as $N_{r}=N_{c}=8$. The network studied thus involves 4096 neurons (8×8 hypercolumns × 64 channels of sparse features) to process a 32×32 image input. This represents a 4-times overcomplete representation. To evaluate whether the results depend on the number of filters, we also tested models with 128 sparse code filters, which, with 9192 neurons, constitute a model with an 8-times overcomplete representation (Fig A3 and Sect C in S1 Text).

### 4.2. Common parameter settings of the model

The network we used is a general V1 network, with most of the model’s parameters being rather standard. The time constant of excitatory neurons ($\tau_{e}=40$) double that of inhibitory neurons ($\tau_{i}=20$) [44]. The local extent of surround inhibition and excitation is also well understood, based on the spatial extent of surround inhibition [45]. Here, we set the radius for mutual facilitation among excitatory neurons to be *R*_*e*_ = 2, and the radius for surround suppression is *R*_*i*_ = 1. The initial weight of plastic excitatory-excitatory connections is *w*_*ee*_ = 5. We set the synaptic time constant $\tau_{w}$ of both the BCM rule and the general Hebbian rule to be 2*e*9, and the time constant of the moving average $\tau_{\xi }$ in the BCM rule to be 2*e*7. For the BCM rule, the initial firing rate threshold for each neuron was set to its average response magnitude, calculated across all stimuli and time steps in the pre-trained network. The absolute strength of the inhibitory surround, *w*_*ie*_, is the main free parameter that was tuned for model stability. We performed a parameter sweep to understand the impact of this parameter on the familiarity effects (sharpening of tuning curves and familiar suppression) under different surround inhibition configurations (Fig A1 and Sect A in S1 Text).

### 4.3. Familiarity effects experiment (Fig 2)

The network was trained for 80 epochs using a set of 25 natural images from the CIFAR-100 dataset. Within each epoch, every image was presented for 300 ms. To prevent inter-stimulus interference, the network’s response $r^{e},r^{i}$ were reset to zero before the presentation of each image. To accelerate learning, the feedforward input during training was scaled by a factor of 30. We set *w*_*ie*_ = 20 for this experiment.

After every 8 epochs of training, the network’s performance was probed. In these tests, the network’s response to the 25 familiar (trained) images was recorded. For each test image, the steady-state response was determined by simulating the network dynamics until convergence and then averaging over the final 20 ms.

To quantify familiarity effects, we analyzed the steady-state responses of the excitatory neuron, $r^{e}$. We calculated a suppression index (SI) for each neuron *i* using the following formula:

$$\text{SI}=\frac{r_{i}^{\text{post}}-r_{i}^{\text{pre}}}{r_{i}^{\text{post}}+r_{i}^{\text{pre}}},$$ (8)

where $r_{i}^{\text{before}}$ and $r_{i}^{\text{after}}$ represent the neuron’s average response to a stimulus set. Before and after training, for each neuron, the SI was computed for every stimulus and then averaged across the entire training set. The distribution of indices is plotted in Fig 2B, where we excluded the non-responsive neurons in pre- or post-training stages.

To measure tuning curve sharpening, we quantified two metrics for each neuron across the stimulus set: its peak firing rate and its lifetime sparsity. The lifetime sparsity of a neuron’s tuning curve was calculated using the index proposed by Vinje and Gallant [46]:

$$S=\frac{n}{n-1}(1-\frac{(\sum_{j}r_{j}/n{)}^{2}}{\sum_{j}(r_{j}^{2}/n)}),$$ (9)

where *r*_*j*_ is the neuron’s response to the *j*-th stimulus out of *n* total stimuli. A value of *S* approaching 1 indicates high lifetime sparsity, meaning the neuron responds selectively to only a small fraction of stimuli, a key feature of a sharpened tuning curve [47]. The relative change of the metric was calculated as (Post−Pre)/(Post+Pre).

### 4.4. Familiarity training experiment to test the Manifold Transform Hypothesis (Fig 3, S1 Fig)

The stimulus set was based on 5 target images randomly selected from the CIFAR-100 dataset and converted to grayscale. Noisy variants of each target image, *l*, were generated by replacing a specified percentage of pixels, n%, with new values drawn from a uniform distribution, ε~𝒰(0,1), defining the conditional stimulus distribution p(x|l,n).

The network was trained on this stimulus set for 350 epochs. In each epoch, the images were presented in a randomized order, with each stimulus shown for 300 ms. To prevent inter-stimulus interference, the network’s activity rates ($r^{e},r^{i}$) were reset to zero prior to each presentation. To accelerate learning, the feedforward input was scaled by a factor of 30. We set *w*_*ie*_ = 30 for this experiment.

The network’s performance was evaluated every 10 epochs using all images in the training set. For each test image, a response fixed point was determined by simulating the network dynamics until convergence and then averaging the excitatory population activity over the final 20 ms. In Fig 3E, we averaged $R^{lev}$ and the $R^{res}$ over the noise pattern *k*. In Fig 3F, 3G, we computed the linear regression and Pearson correlation coefficient of the averaged $R^{lev}$ and the $R^{res}$ over noise pattern, noise level, and target images, with familiarity effects metrics calculated as in Sect 4.3. We set SI for the pre-training network as 0.

### 4.5. Manifold transform via local linear dynamics

The representation manifold ${ℳ}_{r^{*}}$ is implicitly defined by the fixed-point equation of the recurrent circuit:

$$G(𝐫,\alpha (x))=𝐫-\sigma (W𝐫+\alpha (x));\;\text{where }G({𝐫}^{*},\alpha (x))=0.$$ (10)

Here, $𝐫=[{r_{e}}^{⊤};\;{r_{i}}^{⊤}{]}^{⊤}$ is the full network state, and $W=[W_{ee},W_{ei};W_{ie},0]$ (We use “,” to separate column, “;” to separate row) is the block connectivity matrix. To analyze the local geometry of this manifold, we derive a first-order approximation for the squared Euclidean distance, $\langle \|\Delta 𝐫{\|}^{2}\rangle$, between nearby points.

For a small change in the input sparse code, Δα, the corresponding displacement on the manifold, Δ𝐫, can be approximated by a first-order Taylor expansion:

$$\Delta 𝐫\approx (\frac{\partial \;r^{*}}{\partial \;\alpha })\Delta \alpha :=J({𝐫}^{*})\Delta \alpha ,$$ (11)

where the Jacobian matrix $J({𝐫}^{*})$ is given by:

$$J=\underset{\text{recurrent gain, }M}{\underbrace{(I-\Sigma^{\prime }W{)}^{-1}}}\;\Sigma^{\prime }.$$ (12)

Here, $\Sigma^{\prime }$ is a diagonal matrix with elements $\Sigma_{ii}^{\prime }=\sigma^{\prime }(W{𝐫}^{*}\;+\;\alpha (x){)}_{i}$, measuring the local sensitivity of each neuron. The term $M:={\left(I-\Sigma^{\prime }W\right)}^{-1}$ is the recurrent gain matrix, which captures how the recurrent circuitry amplifies or suppresses feedforward inputs in the linearized system.

The average squared distance can now be expressed as $\langle \|\Delta 𝐫{\|}^{2}\rangle =\langle \Delta \alpha^{⊤}J^{⊤}J\;\Delta \alpha \rangle$. To understand how recurrent dynamics shape this distance, we analyze *M* through its eigendecomposition, $M=\sum_{i}\mu_{i}w_{i}v_{i}^{⊤}$, where $\mu_{i}$ are the eigenvalues and $w_{i},v_{i}$ are the right and left eigenvectors, respectively. Substituting the eigendecomposition of the recurrent gain matrix into the average squared distance formula yields:

$$\langle \|\Delta 𝐫{\|}^{2}\rangle =\langle \Delta \alpha^{⊤}J^{⊤}J\;\Delta \alpha \rangle$$ (13)

$$=\langle \sum_{ij}\mu_{i}\mu_{j}(\underset{{\tilde{v}}_{i}^{⊤}}{\underbrace{v_{i}^{⊤}\Sigma^{\prime }}}\Delta \alpha )(\underset{{\tilde{v}}_{j}^{⊤}}{\underbrace{v_{j}^{⊤}\Sigma^{\prime }}}\Delta \alpha )(w_{i}^{⊤}w_{j})\rangle$$ (14)

$$=\langle {𝐩}^{⊤}G\;𝐩\rangle .$$ (15)

This equation reveals that the geometry is determined by the interplay of two key factors. The first is captured by the vector **p**, where each element $p_{i}=\mu_{i}({\tilde{v}}_{i}^{⊤}\Delta \alpha )$ represents the input change Δα projected onto an input filter ${\tilde{v}}_{i}$ and scaled by the recurrent gain $\mu_{i}$. The second is the Gram matrix *G*, with elements $G_{ij}=w_{i}^{⊤}w_{j}$, which captures the geometric overlap between the output patterns (right eigenvectors *w*_*i*_ and *w*_*j*_) of the recurrent gain modes. Thus, the manifold’s local geometry depends on both how the linearized dynamics modulate certain input patterns and how the output patterns interact with each other.

To simplify the mode interactions, we diagonalize the Gram matrix, $G=\sum_{k}\lambda_{k}q_{k}q_{k}^{⊤}$, which reveals a set of orthogonal collective modes. Substituting this back into the distance calculation yields the final expression (Eq 6):

$$\langle \|\Delta 𝐫{\|}^{2}\rangle =\sum_{k}\lambda_{k}\sum_{ij}(q_{k}{)}_{i}\langle p_{i}p_{j}\rangle (q_{k}{)}_{j}$$ (16)

$$=\sum_{k}\lambda_{k}\sum_{ij}(q_{k}{)}_{i}\mu_{i}{\tilde{v}}_{i}^{⊤}\langle \Delta \alpha \;\Delta \alpha^{⊤}\rangle {\tilde{v}}_{j}\mu_{j}(q_{k}{)}_{j}$$ (17)

$$=\sum_{k}\lambda_{k}\;\varphi_{k}^{⊤}\langle \Delta \alpha \;\Delta \alpha^{⊤}\rangle \varphi_{k}.$$ (18)

This equation decomposes the squared distance into contributions from each collective mode *k*. Each term consists of: 1) Spectrum ($\lambda_{k}$): The eigenvalue of the *k*-th collective mode, which acts as its overall amplification gain, and 2) Alignment ($g_{k}:=\varphi_{k}^{⊤}\langle \Delta \alpha \;\Delta \alpha^{⊤}\rangle \varphi_{k}$), which measures how much the input variations are projected along the direction of the mode’s effective input filter, $\varphi_{k}$. This filter, defined as $\varphi_{k}:=\sum_{i}(q_{k}{)}_{i}\mu_{i}{\tilde{v}}_{i}$, is the sum of the input filter of the original recurrent gain mode weighted by the recurrent gain and their contributions to the collective mode *k*.

### 4.6. Linear analysis in the familiarity-trained network (Fig 4)

To analyze the local linear transform, we first numerically computed the collective modes for the network trained in the familiarity association task. For each attractor, we calculated the Jacobian and the corresponding recurrent gain matrix, *M*. We then performed an eigenvalue decomposition on *M* to find its recurrent gain modes. From the right eigenvectors of *M*, we computed the Gram matrix *G* and performed a second eigenvalue decomposition on it to determine the properties of the collective modes. This process yielded the spectrum ($\lambda_{k}$) and effective input filters ($\varphi_{k}$) for each collective mode as derived in the previous sections.

Using these computed modes, we then calculated the linear approximation of the relative distances to probe the local manifold geometry. This was achieved by applying the collective mode distance formula (Eq 6), which requires computing input variation vector Δα along the signal, residual and level directions in the input space. To isolate the effects of local geometry from magnitude, each Δα was normalized to unit length. All results were based on the top 10 collective modes, which consistently had the largest eigenvalues. For Fig 4D, 4E, a mode was included if the magnitude of its change (e.g., in spectrum or alignment) exceeded 10% of the maximum change observed across all modes. We established the orientation of each mode: a mode was assigned as signal-oriented if its pre-training signal alignment exceeded its noise alignment by more than 10% of the maximum observed difference, and vice versa for noise-oriented modes.

### 4.7. Sensitivity of alignment and spectrum change of individual collective mode to the compression (Fig 4)

To quantify how learning-induced changes in the collective mode spectrum ($\lambda_{k}$) and alignment (*g*_*k*_) contribute to noise compression, we performed a sensitivity analysis. First, we defined the linear component of signal/noise distances: $D=\sum_{k}\lambda_{k}g_{k}$. The linear relative distance is $R=D_{\text{noise}}/D_{\text{signal}}$

To understand how changes in the network parameters affect *R*, we performed a first-order Taylor expansion around the pre-training state:

$$\Delta R\approx \frac{\partial R}{\partial D_{\text{signal, pre}}}\Delta D_{\text{signal}}+\frac{\partial R}{\partial D_{\text{noise, pre}}}\Delta D_{\text{noise}}$$ (19)

$$=\frac{D_{\text{signal, pre}}}{D_{\text{signal, pre}}^{2}}\Delta D_{\text{noise}}-\frac{D_{\text{noise, pre}}}{D_{\text{signal, pre}}^{2}}\Delta D_{\text{signal}}.$$ (20)

From this, the necessary and sufficient condition for learning to improve compression (ΔR<0) is that the fractional change in signal distancce must exceed the fractional change in noise distance: $\Delta D_{\text{signal}}/D_{\text{signal, pre}}>\Delta D_{\text{noise}}/D_{\text{noise, pre}}$.

We define the fractional change as $\tilde{D}$, and decompose it into components related to the change in spectrum, $\Delta \lambda_{k}$, and the change in alignment, $\Delta g_{k}$. The change in linear distance is $\Delta D=\sum_{k}(\lambda_{k,\text{pre}}\Delta g_{k}\;+\;g_{k,\text{pre}}\Delta \lambda_{k}\;+\;\Delta \lambda_{k}\Delta g_{k})$. Focusing on the linear terms and omitting the interaction term, the fractional change is approximately:

$$\tilde{D}\approx \frac{\sum_{k}(\lambda_{k,\text{pre}}\Delta g_{k}+g_{k,\text{pre}}\Delta \lambda_{k})}{\sum_{j}\lambda_{j,\text{pre}}g_{j,\text{pre}}}.$$ (21)

The sensitivity of this quantity with respect to a change in the spectrum of mode *k* is the fraction of pre-trained distance contributed by that mode’s pre-training alignment:

$$\frac{\partial \tilde{D}}{\partial \Delta \lambda_{k}}=\frac{g_{k,\text{pre}}}{\sum_{j}\lambda_{j,\text{pre}}g_{j,\text{pre}}}.$$ (22)

This yields three sensitivity values for the spectrum change, each with respect to fractional change in signal distance, noise level distance, and residual noise distance, respectively. We derived a combined sensitivity measure by averaging these three values. The absolute magnitude of this average was then normalized by the maximum observed magnitude to produce the normalized sensitivity plotted in Fig 4E.

Similarly, the sensitivity with respect to a change in alignment is the fraction of pre-trained distance contributed by that mode’s pre-training spectrum:

$$\frac{\partial \tilde{D}}{\partial \Delta g_{k}}=\frac{\lambda_{k,\text{pre}}}{\sum_{j}\lambda_{j,\text{pre}}g_{j,\text{pre}}}.$$ (23)

This yields distinct sensitivity values for signal, noise level, and residual noise alignments to the fractional changes of signal, noise level, and residual noise distances. We derived a combined noise sensitivity by averaging the latter two. Each value was then converted to its absolute magnitude and subsequently normalized by the maximum magnitude to derive the normalized sensitivities plotted in Fig 4E.

## Supporting information

S1 Fig Additional visualization of the neural manifold in the familiarity association experiment. **(A)** Trajectories of different noise levels correspond to an example image in the model. The trajectory is averaged across noisy image samples. The black arrow indicates the direction along which the noise level changes (denoted as the image-to-noise axis). Cross: trial start; Dots: trial end. **(B)** Each dot (ellipse) of a particular color represents a sample noise image (or the covariance of the set of sample images) of the target image at a particular noise level. Each red cross represents the mean of clusters at each noise level. **(C)** Each dot represents the cluster mean of a target image at a specific noise level. The five clusters of dots correspond to the five target images and their noise variants, with color indicating the noise level. Each ellipse represents the covariance of the samples of the five targeted images at a particular noise level. Each red cross represents the average of cluster means of the same noise level. For **B** and **C**, the dots and circles, derived from real test images, correspond to the blue and orange cone, respectively, depicted in the schematic illustrations in Fig 3D.
(TIF)

S1 Text Additional results. (PDF)
