# Supplementary figures and images for: Manifold transform by recurrent cortical circuit enhances robust encoding of familiar stimuli
Source: PLoS Comput Biol. 2025 Oct 24;21(10):e1013587. doi: 10.1371/journal.pcbi.1013587 (PMC12551896; doi:10.1371/journal.pcbi.1013587)

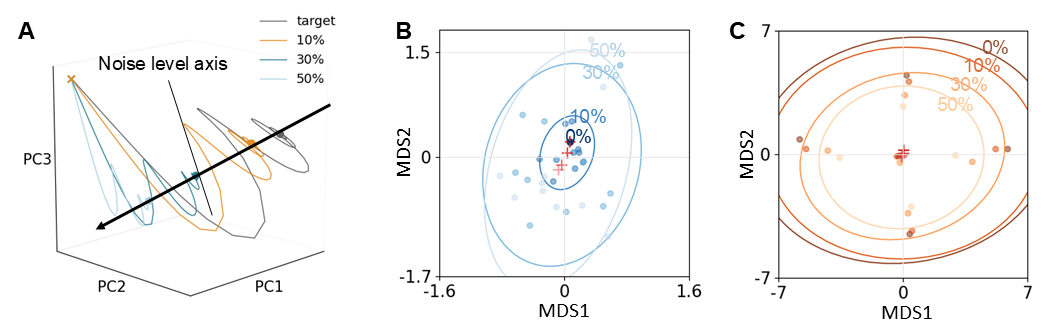

Supplement: S1 Fig — (A) Trajectories of different noise levels correspond to an example image in the model. The trajectory is averaged across noisy image samples. The black arrow indicates the direction along which the noise level changes (denoted as the image-to-noise axis). Cross: trial start; Dots: trial end. (B) Each dot (ellipse) of a particular color represents a sample noise image (or the covariance of the set of sample images) of the target image at a particular noise level. Each red cross represents the mean of clusters at each noise level. (C) Each dot represents the cluster mean of a target image at a specific noise level. The five clusters of dots correspond to the five target images and their noise variants, with color indicating the noise level. Each ellipse represents the covariance of the samples of the five targeted images at a particular noise level. Each red cross represents the average of cluster means of the same noise level. For B and C, the dots and circles, derived from real test images, correspond to the blue and orange cone, respectively, depicted in the schematic illustrations in Fig 3D. (TIF) [file pcbi.1013587.s001.tif]
