# Supplementary material for: Manifold transform by recurrent cortical circuit enhances robust encoding of familiar stimuli
Source: PLoS Comput Biol. 2025 Oct 24;21(10):e1013587. doi: 10.1371/journal.pcbi.1013587 (PMC12551896; doi:10.1371/journal.pcbi.1013587)
Supplement: S1 Text — (PDF) [file pcbi.1013587.s002.pdf]

A Impacts of the surround inhibition on familiarity effects

While inhibitory connectivity in the cortex is typically distance-dependent [1, 2, 3], our primary model employs spatially uniform inhibition for simplicity. To validate this choice, we compared the Hebbian model's performance against a network with spatially-dependent inhibition, where weights (w_{ie}) followed a Gaussian profile (up to $\sigma = 1$). The Gaussian profile models a distance-dependent inhibition, where strength decays with distance from the neighborhood's center, while the total inhibitory strength within the neighborhood is preserved. We also investigated the effect of inhibitory strength (w_{ie}) on the familiarity effects for both inhibition profiles. We found that the network with uniform inhibition became unstable at weak strengths, whereas the Gaussian model remained stable across the tested range. In the uniform network, familiarity suppression and tuning curve sharpening emerged when $w_{ie} > 20$, with the magnitude of these effects diminishing as w_{ie} was increased further. In contrast, these effects appeared earlier in the Gaussian network, at $w_{ie} = 15$. Although both models produced the key phenomena, the Gaussian profile resulted in a stronger average suppression that was concentrated in a smaller proportion of the neuronal population. Similarly, tuning sharpening was observed in fewer neurons compared to the uniform inhibition model.

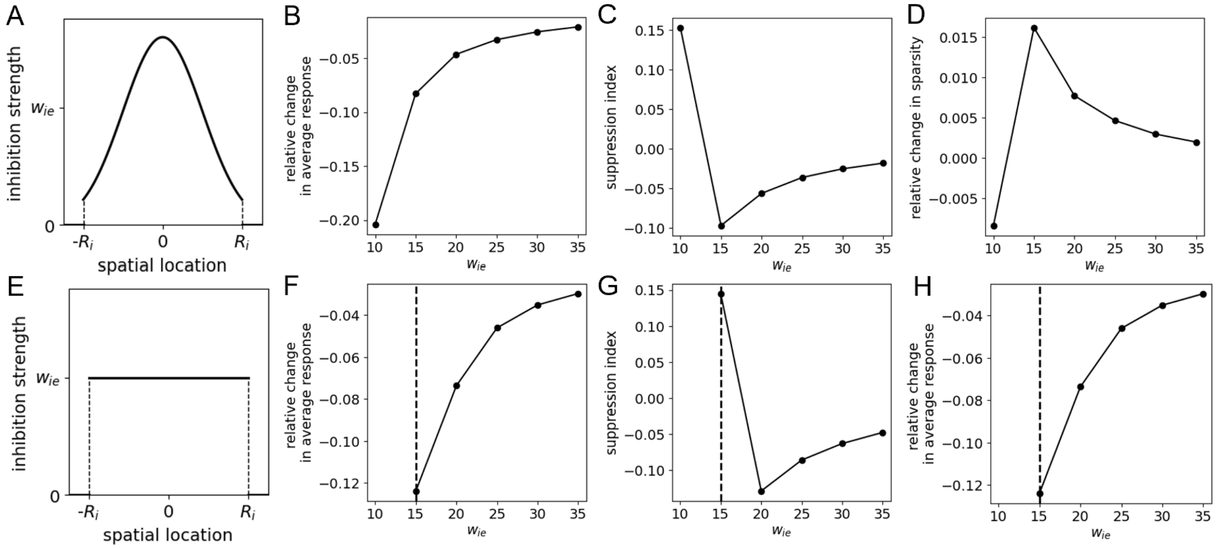

Figure A1: **Impacts of surround inhibition on familiarity effects A**: Illustration of Gaussian inhibition. The X-axis is the spatial location, where the target inhibitory neuron is at 0, and its inhibitory neighborhood spans the range from $-R_i$ to R_i. The Y-axis is the inhibition strength. In Gaussian inhibition, the E to I strength varies around the initial strength w_{ie} in a distance-dependent manner. **B-D** familiarity effects metrics (relative change in the average response, suppression index, and relative change in lifetime sparsity) as a function of surround inhibition strength (w_{ie}), for Gaussian inhibition profile. **E**: Same as in A but for uniform inhibition, where all E to I strengths equal the initial weight w_{ie}. **F-H**: familiarity effects metrics (relative change in the average response, suppression index, and relative change in lifetime sparsity) as a function of surround inhibition strength (w_{ie}), for Gaussian inhibition profile.

B Additional results on manifold compression

In our original experiment, Hebbian learning remains active when noisy versions of the images are presented. This setup mimics realistic neurophysiological conditions. In the simulation experiment, the target stimuli are presented more frequently (30 times more) than their noisy counterparts in each epoch, but learning is nonetheless ongoing during all stimulus exposures. However, this leaves open the question: is exposure to the noisy images necessary for inducing the compression of the noise-variant manifold? To address this, we performed a control experiment in which the network was solely trained on the clean images. We found that compression of the noise dimensions still occurred (Fig S3A). This suggests that intense familiarity training on clean images alone is sufficient to drive manifold compression of noise-induced variability.

For completeness, we also tested the performance of the BCM rule on the manifold transform, using the same experimental setting as in the main text. Fig. S3B shows that the BCM rule results in a weaker compression effect due to the rebound at the later stage of training. However, it is possible that this difference can be attributed to suboptimal hyperparameter settings, especially the change speed of the sliding threshold.

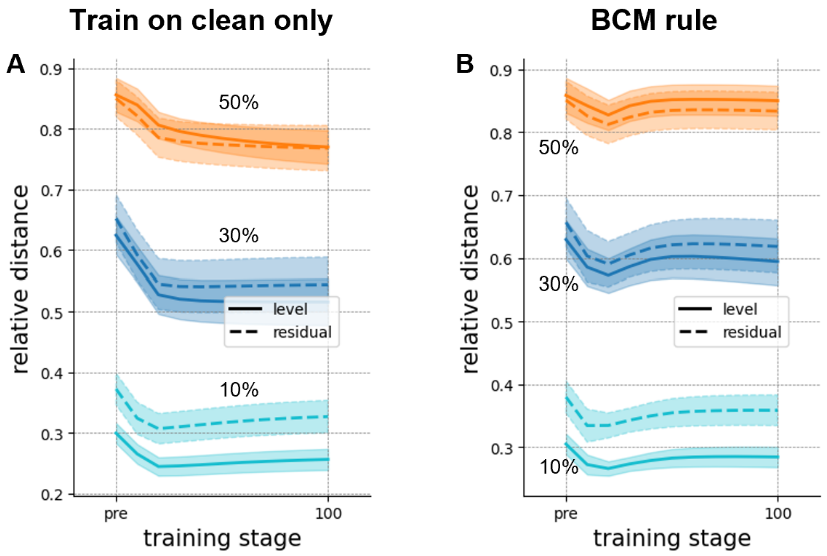

Figure A2: **Additional results on manifold compression (A)** Qualitatively similar learning curves of relative level and residual distances across all noise levels as in Fig 3E, but for the network trained only on clean images. Ribbons indicate standard deviation. **(B)** Learning curves for relative level and residual distances across all noise levels for the network trained with the BCM rule. Ribbons indicate standard deviation. The learning dynamic is slightly different than Fig 3E, but the relative distances still show net decreases.

C Effect of filter number on familiarity effects and manifold compression

The network studied involves 4096 neurons (8×8 hypercolumns $\times$ 64 channels of sparse features) to process a 32×32 image input. This represents a 4-times overcomplete representation. We found that doubling the number of feature channels in the network, i.e., 8-times overcomplete, produces very similar results in familiarity suppression, sparsification of population code as well as manifold compression effects (Fig S4), suggesting that our findings are not specific to the selection of sparse code feature channels and are quite robust against changes in hyperparameters in the model.

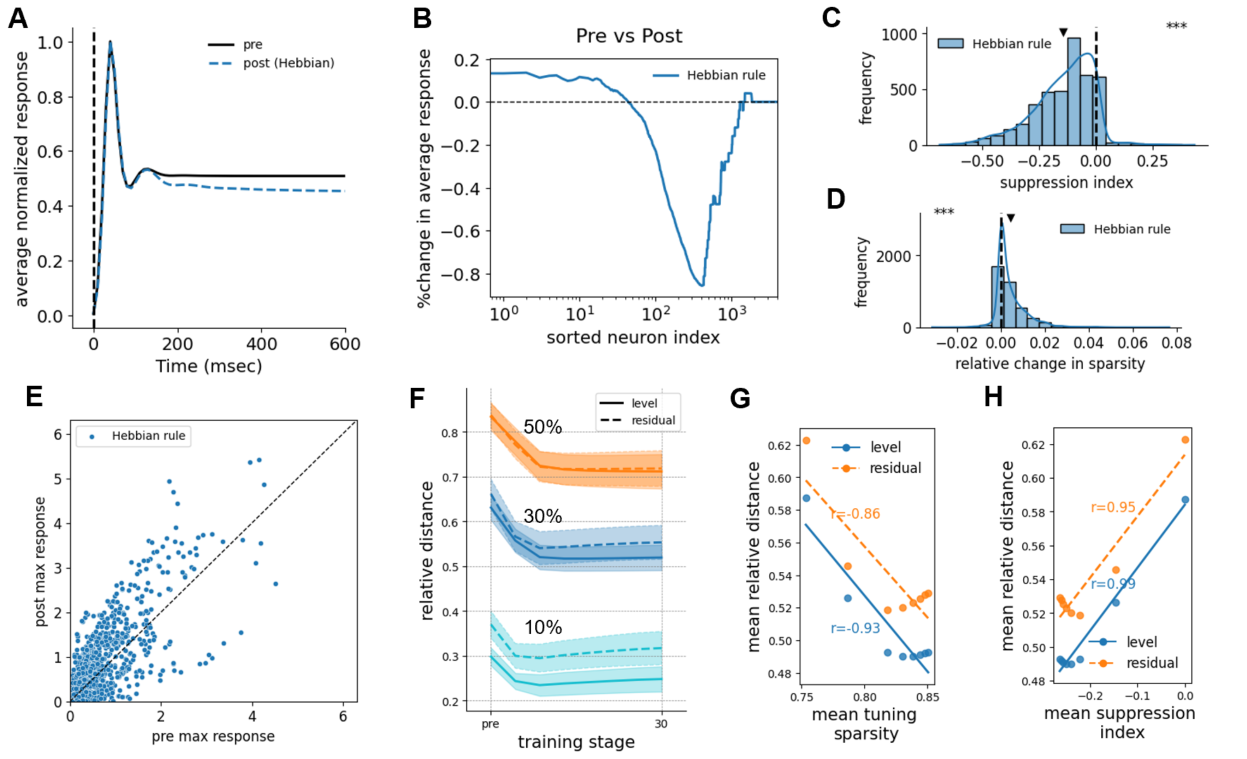

Figure A3: **Additional results for the 128 filter network.** All panels shown are analogous to the analyses in Figures 2 and 3. **(A)** Average population response (r/r_{max}) suppression during familiarity training. **(B)** Stimulus-averaged change in population tuning curves, with neurons sorted by descending response (x-axis on log scale). **(C, D)** Population histograms of the neuronal suppression index (C) and relative change in lifetime sparsity (D). **(E)** Scatter plot of maximum neuronal response before versus after training. **(F)** Learning curves for relative level and residual distances across all noise levels. Ribbons indicate standard deviation. **(G, H)** Reverse correlation between relative distances and neuronal selectivity (G) or suppression magnitude (H). Solid lines are linear regression fits with Pearson's r noted. *For (C) and (D), triangle markers indicate the mean (***: $p < 0.001$, one-sided t-test against 0).*

D Compression of variants manifold results in better concept discriminability in neurons of higher-level visual area

While our network is modeled after the primary visual cortex (e.g., V1, V2), the function we investigate - the use of manifold transforms to achieve robust representations against noise, rotation, and other variations — is a hallmark of processing typically attributed to higher visual areas like the inferotemporal (IT) cortex. This leads to the question of how manifold transforms in the early visual areas impact subsequent stages of the visual hierarchy. To address this, we simulated a subsequent processing layer and evaluated its ability to perform concept discrimination based on the representation of the recurrent circuit.

We modeled a readout layer of five neurons in the higher-order visual area (e.g., IT), each intended to represent one of the five visual concepts. These neurons received input from all excitatory neurons in the recurrent circuit, and their synaptic weights ($\mathbf{w}_i$) were trained for 10 epochs using a competitive learning rule based on K-means clustering. For each input representation $\mathbf{r}$ from the full set of clean and noisy images, the winning neuron i (the one with the minimum cosine distance between its weight vector $\mathbf{w}_i$ and $\mathbf{r}$) updated its weights according to: $\mathbf{w}_{i,t+1} = \mathbf{w}_{i,t} + \eta \ (\mathbf{r} - \mathbf{w}_{i,t})$. Crucially, to assess the impact of the familiarity training at the lower level, the learning of readout neurons was performed independently on sets of recurrent representations at different stages (specifically, epochs below 30, where the compression effect was strongest).

We found that the resulting representations of the readout layer formed distinct clusters for each visual concept. We quantified the separability of these clusters by computing the sensitivity index (d') between each cluster and its nearest neighbor: $d' = \sqrt{(\mu_i - \mu_j)^\top \ (0.5(\Sigma_i + \Sigma_j))^{-1}(\mu_i - \mu_j)}$. The results demonstrate that as the lower-level recurrent network underwent familiarity training, its representations enabled progressively better higher-level concept discrimination, evidenced by a significant increase in the d' index (Fig. S5A). This enhanced discriminability was qualitatively confirmed by t-SNE embeddings of the higher-level representations (Fig S5B), which showed that the neuron at the readout layer can encode each cluster and exhibit invariance thanks to the invariance achieved by the recurrent circuits in the preceding layer.

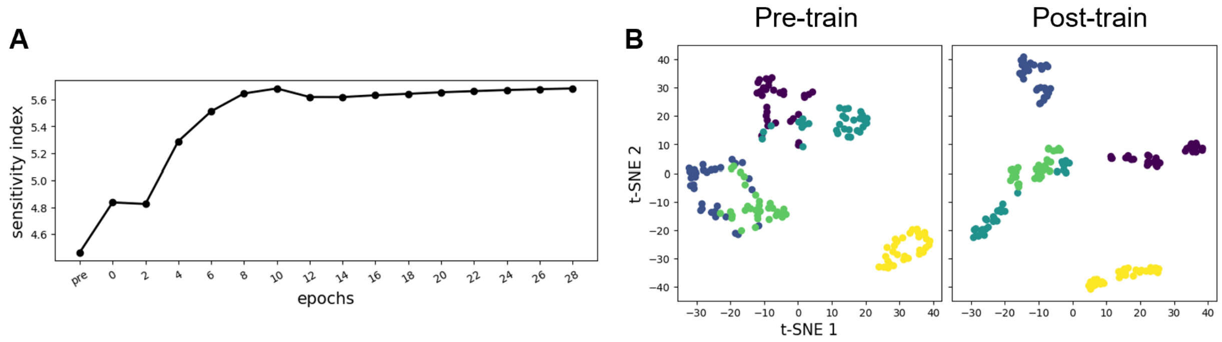

Figure A4: **Higher-level impact of manifold compression (A)**: The average sensitivity index of higher-level representation as a function of familiarity training epoch of the recurrent circuit. The concept discriminability of the higher-level neurons increases as the familiarity training proceeds. **(B)**: t-SNE embedding of the higher-level representation before training and after training (epoch 28). Each color represents a different visual concept. After training, the higher-level representation becomes more clustered with visual concepts.

References

[1] Hennequin G, Ahmadian Y, Rubin DB, Lengyel M, Miller KD. The dynamical regime of sensory cortex: stable dynamics around a single stimulus-tuned attractor account for patterns of noise variability. Neuron. 2018;98(4):846–860.

[2] Rubin DB, Van Hooser SD, Miller KD. The stabilized supralinear network: a unifying circuit motif underlying multi-input integration in sensory cortex. Neuron. 2015;85(2):402–417.

[3] Mosheiff N, Ermentrout B, Huang C. Chaotic dynamics in spatially distributed neuronal networks generate population-wide shared variability. PLOS Computational Biology. 2023;19(1):e1010843.
